# Neoadjuvant chemotherapy drives intratumoral T cells toward a proinflammatory profile in pancreatic cancer

**DOI:** 10.1172/jci.insight.152761

**Published:** 2022-11-22

**Authors:** Max Heiduk, Ioana Plesca, Jessica Glück, Luise Müller, David Digomann, Charlotte Reiche, Janusz von Renesse, Rahel Decker, Christoph Kahlert, Ulrich Sommer, Daniela E. Aust, Marc Schmitz, Jürgen Weitz, Lena Seifert, Adrian M. Seifert

**Affiliations:** 1 Department of Visceral, Thoracic and Vascular Surgery, University Hospital Carl Gustav Carus, Technische Universität Dresden, Dresden, Germany.; 2 National Center for Tumor Diseases, Dresden, Germany; German Cancer Research Center, Heidelberg, Germany; Faculty of Medicine and University Hospital Carl Gustav Carus, Technische Universität Dresden, Dresden, Germany; and Helmholtz-Zentrum Dresden-Rossendorf, Dresden, Germany.; 3 Institute of Immunology, Faculty of Medicine Carl Gustav Carus, Technische Universität Dresden, Dresden, Germany.; 4 German Cancer Consortium, Partner Site Dresden, German Cancer Research Center, Heidelberg, Germany.; 5 Institute of Pathology, Faculty of Medicine Carl Gustav Carus, and; 6 National Center for Tumor Diseases, Biobank Dresden, University Hospital Carl Gustav Carus, Technische Universität Dresden, Dresden, Germany.

**Keywords:** Immunology, Oncology, Cancer, Cytokines, T cells

## Abstract

**BACKGROUND:**

Pancreatic ductal adenocarcinoma (PDAC) has a dismal prognosis. At diagnosis, only 20% of patients with PDAC are eligible for primary resection. Neoadjuvant chemotherapy can enable surgical resection in 30%–40% of patients with locally advanced and borderline resectable PDAC. The effects of neoadjuvant chemotherapy on the cytokine production of tumor-infiltrating T cells are unknown in PDAC.

**METHODS:**

We performed multiplex immunofluorescence to investigate T cell infiltration in 91 patients with PDAC. Using flow cytometry, we analyzed tumor and matched blood samples from 71 patients with PDAC and determined the frequencies of T cell subsets and their cytokine profiles. Both cohorts included patients who underwent primary resection and patients who received neoadjuvant chemotherapy followed by surgical resection.

**RESULTS:**

In human PDAC, T cells were particularly enriched within the tumor stroma. Neoadjuvant chemotherapy markedly enhanced T cell density within the ductal area of the tumor. Whereas infiltration of cytotoxic CD8^+^ T cells was unaffected by neoadjuvant chemotherapy, the frequency of conventional CD4^+^ T cells was increased, and the proportion of Tregs was reduced in the pancreatic tumor microenvironment after neoadjuvant treatment. Moreover, neoadjuvant chemotherapy increased the production of proinflammatory cytokines by tumor-infiltrating T cells, with enhanced TNF-α and IL-2 and reduced IL-4 and IL-10 expression.

**CONCLUSION:**

Neoadjuvant chemotherapy drives intratumoral T cells toward a proinflammatory profile. Combinational treatment strategies incorporating immunotherapy in neoadjuvant regimens may unleash more effective antitumor responses and improve prognosis of pancreatic cancer.

**FUNDING:**

This work was supported by the Jung Foundation for Science and Research, the Monika Kutzner Foundation, the German Research Foundation (SE2980/5-1), the German Cancer Consortium, and the Faculty of Medicine Carl Gustav Carus, Technische Universität Dresden.

## Graphical abstract

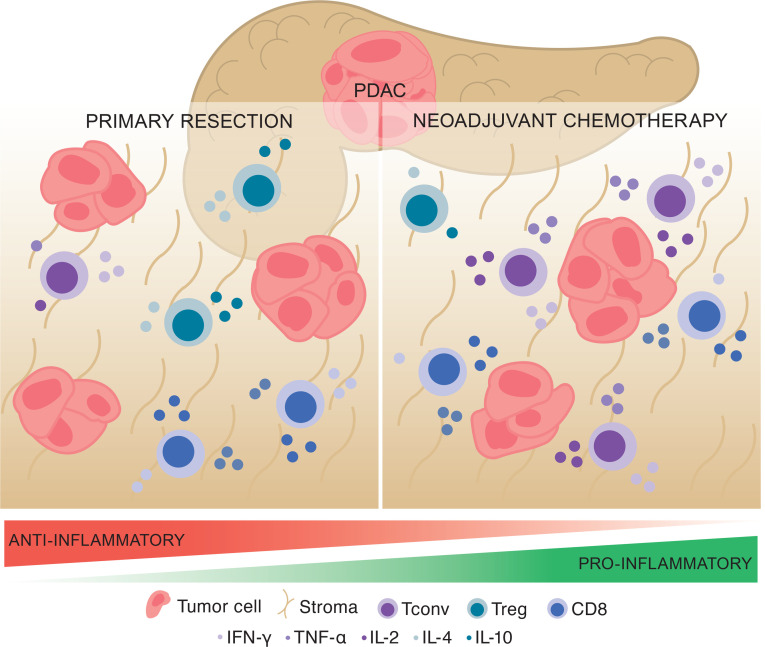

## Introduction

Pancreatic ductal adenocarcinoma (PDAC) has a dismal prognosis, with only 10% of patients surviving 5 years after diagnosis (1). Surgical resection is the only potentially curative treatment; however, patients are often diagnosed at an advanced disease stage. At diagnosis only 20% of patients with PDAC are eligible for primary resection (PR) (2). Neoadjuvant chemotherapy (NEO) can enable surgical resection in 30%–40% of patients with locally advanced and borderline resectable PDAC (3–6). Even after complete tumor resection, 80% of patients develop tumor recurrence and die within 2 years (7, 8). Overall, patient outcomes have not improved significantly with current therapies over the past years (9). Accumulating evidence indicates that the immune system makes a crucial contribution to the antitumor effects of chemotherapy (10–12). Beyond tumor cell–specific factors that determine cytotoxic and immune responses, the functional state of the host immune system has a relevant effect on patient prognosis. PDAC is characterized by a heterogeneous and mostly immunosuppressive immune infiltrate. T cells are the most prevalent immune cell type, with intermediate to high levels of T cell infiltration in PDAC (13, 14). The tumor-infiltrating lymphocyte composition and spatial distribution defined distinct immunological PDAC subtypes that correlated with patient prognosis (15, 16). The presence of intratumoral CD8^+^ T cells as well as the polarization of conventional CD4^+^ T (Tconv) cells toward a Th1 phenotype were both associated with prolonged survival in human PDAC, whereas Th2 cells promoted tumor progression in murine pancreatic cancer (15, 17–19). Furthermore, high levels of immunosuppressive Tregs in the peripheral blood and tumor stroma were associated with poor clinical outcomes in human PDAC (20, 21). These data were almost entirely derived from limited immunohistochemical analyses, while functional studies are lacking. Analysis of rare long-term survivors of PDAC revealed persistence of T cell clones specific to tumor antigens (22). Patients with PDAC with high T cell infiltration and neoantigen qualities promoting T cell responses had improved survival (22, 23). The effect of NEO on the cytokine profile of PDAC-infiltrating T cells is unknown. In this study, we analyzed freshly isolated T cells from blood and matched tumor specimens from patients with PDAC who were either primarily resected or treated with NEO prior to surgery.

## Results

### Neoadjuvant chemotherapy increases the frequency of PDAC-infiltrating CD4^+^ Tconv cells and reduces the proportion of Tregs.

**Figure 1:**
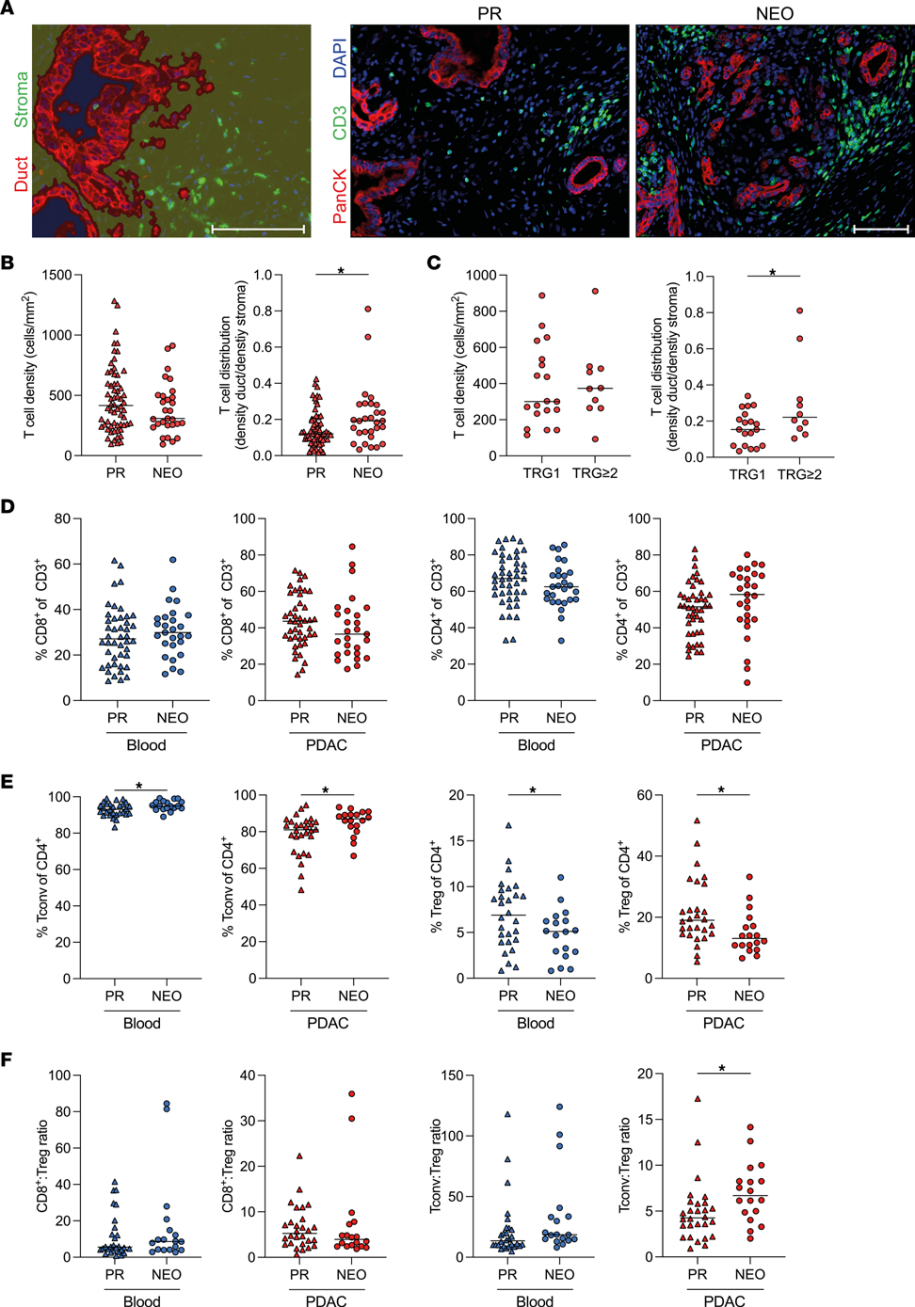
Neoadjuvant chemotherapy increases the frequency of tumor-infiltrating CD4^+^ Tconv cells and reduces the proportion of Tregs. (**A**) Paraffin-embedded human pancreatic ductal adenocarcinoma (PDAC) specimens from patients who were primary resected (PR) or received neoadjuvant chemotherapy (NEO) prior to surgery were stained for DAPI (blue), PanCK (red), and CD3 (green). Representative tissue segmentation and multiplex immunofluorescence images are shown. Scale bar: 100 μm. (**B**) Quantification of T cell density in whole PDAC specimens and distribution (density duct/density stroma) of patients who were PR (*n* = 62) or received NEO (*n* = 29). (**C**) T cell density and distribution according to tumor regression grade (TRG) of patients who received NEO with minor response (TRG1, *n* = 19) and moderate or major response (TRG ≥ 2, *n* = 10). (**D**) Flow cytometric analysis of circulating (blue) and matched PDAC-infiltrating leucocytes (red) from patients with PDAC who were PR and who received NEO. Quantification of CD8^+^ (*n* = 71; left) and CD4^+^ (*n* = 71; right) among all CD3^+^ T cells and (**E**) conventional CD4^+^ T (Tconv; CD4^+^FOXP3^–^; *n* = 46; left) cells and Tregs (CD4^+^FOXP3^+^; *n* = 46; right) among all CD4^+^ T cells. (**F**) Ratio of CD8^+^ T cells to Tregs (*n* = 46; left) and CD4^+^ Tconv cells to Tregs (*n* = 46; right). Each point represents data from 1 patient. Medians are shown as horizontal lines. Unpaired 2-tailed *t* test. *P < 0.05.

To evaluate the effects of NEO on T cell infiltration in PDAC, we performed multiplex immunofluorescence for DAPI, PanCK, and CD3 on tumor specimens from 62 patients who were primary resected (PR) and 29 patients with PDAC (Supplemental Tables 1 and 2; supplemental material available online with this article; https://doi.org/10.1172/jci.insight.152761DS1) who received neoadjuvant chemotherapy (NEO) prior to resection (Figure 1A). The intratumoral density of T cells was highly variable across tumors but unaffected by neoadjuvant treatment (Figure 1B). However, there was a significant difference in T cell distribution between the PR and NEO cohort (Figure 1B). Whereas PDAC-infiltrating T cells generally tended to reside in the stromal area, NEO increased T cell density in the ductal area. Patients with a moderate (tumor regression grade 2 [TRG2]) or major (TRG3) response showed increased T cell distribution compared with patients with a minor (TRG1) response to NEO (Figure 1C). FOLFOXIRI or FOLFIRINOX treatment increased T cell distribution compared with chemotherapy with gemcitabine and/or nab-paclitaxel (Supplemental Figure 1).

To further characterize different T cell subpopulations, we performed flow cytometry on T cells from the peripheral blood and tumors of 71 patients with PDAC (Supplemental Figure 2 and Supplemental Tables 3 and 4). Neoadjuvant chemotherapy did not alter the frequency of CD8^+^ and CD4^+^ T cells among all T cells (Figure 1D). To further delineate the composition of the CD4^+^ T cell population, we stained for the transcription factor FOXP3 to differentiate between CD4^+^ Tconv cells and Tregs. We found PDAC to be highly infiltrated by Tregs, which account for approximately 20% of all tumor-infiltrating CD4^+^ T cells (Figure 1E). Neoadjuvant chemotherapy markedly reduced the proportion of Tregs among CD4^+^ T cells (Figure 1E), significantly increasing the ratio of CD4^+^ Tconv cells to Tregs (Figure 1F). T cell frequencies did not differ between FOLFOXIRI and FOLFIRINOX compared with gemcitabine and/or nab-paclitaxel treatment (Supplemental Figure 3).

### PDAC-infiltrating CD4^+^ T cells have enhanced proinflammatory cytokine production after NEO.

**Figure 2:**
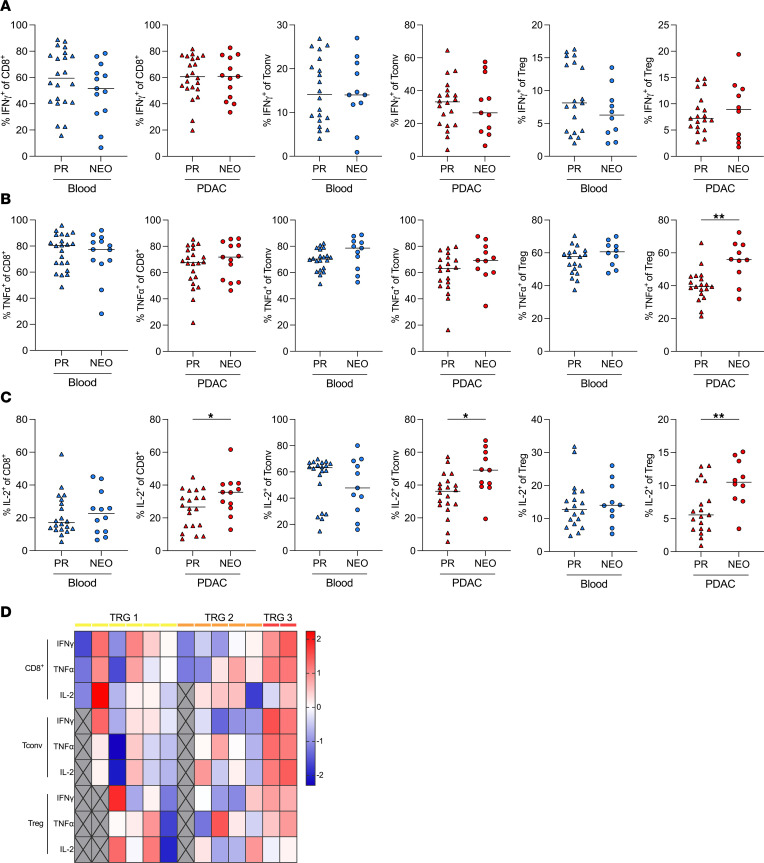
PDAC-infiltrating CD4^+^ T cells have enhanced proinflammatory cytokine production after neoadjuvant chemotherapy. Intracellular cytokine production of circulating and matched PDAC-infiltrating T cells from patients with PDAC. Percentages of (**A**) IFN-γ, (**B**) TNF-α, and (**C**) IL-2 production by CD8^+^ T cells (left), CD4^+^ Tconv cells (middle), and Tregs (right). IFN-γ^+^ and TNF-α^+^ cells of CD8^+^ T cells, *n* = 22 PR, *n* = 13 NEO; of Tconv cells, *n* = 20 PR, *n* = 11 NEO; of Tregs, *n* = 19 PR, *n* = 10 NEO. IL-2^+^ cells of CD8^+^ T cells, *n* = 20 PR, *n* = 12 NEO; of Tconv cells, *n* = 20 PR; *n* = 11 NEO; of Tregs, *n* = 19 PR, *n* = 10 NEO. (**D**) Heatmap depicting the percentage of IFN-γ–, TNF-α–, and IL-2–expressing CD8^+^ T cells, CD4^+^ Tconv cells, and Tregs standardized to *z* score ordered by tumor regression grade (TRG). Missing values are shown in gray. Each point represents data from 1 patient. Medians are shown as horizontal lines. Unpaired 2-tailed *t* test. *P < 0.05; **P < 0.01.

Given the effect of NEO on T cell frequencies, we investigated the T cell cytokine profile using intracellular cytokine staining (Supplemental Figure 4). We analyzed the production of the proinflammatory cytokines IFN-γ, TNF-α, and IL-2 in 22 patients who were PR and 13 patients who received NEO. IFN-γ was mostly produced by CD8^+^ T cells with no detectable difference between blood and tumor CD8^+^ T cells and irrespective of neoadjuvant treatment (Figure 2A). PDAC-infiltrating CD4^+^ Tconv cells produced higher levels of IFN-γ (PR, 28.9% ± 4.7% and NEO, 34.1% ± 7.5%, respectively) compared with CD4^+^ Tconv cells from matched blood (PR, 13.3% ± 4.7% and NEO, 13.3% ± 4.4%, respectively; Figure 2A). After NEO, IFN-γ expression by CD4^+^ Tconv cells and Tregs remained unchanged (Figure 2A). Notably, tumor-infiltrating Tregs showed increased TNF-α production in the NEO cohort compared with the PR cohort (Figure 2B). PDAC-infiltrating CD8^+^ T cells in the NEO cohort produced more IL-2 than corresponding circulating CD8^+^ T cells and tumor-infiltrating T cells in the PR cohort. Both CD4^+^ Tconv cells and Tregs from the tumor expressed less IL-2 than corresponding circulating T cells in the PR cohort. Neoadjuvant chemotherapy increased IL-2 production by all tumor-infiltrating T cell subsets (Figure 2C). Notably, response to NEO was associated with the cytokine profile of tumor-infiltrating T cells. The 2 patients with a major response (TRG3) showed the highest expression of IFN-γ and TNF-α (Figure 2D).

### PDAC-infiltrating T cells have reduced antiinflammatory cytokine production after NEO.

**Figure 3:**
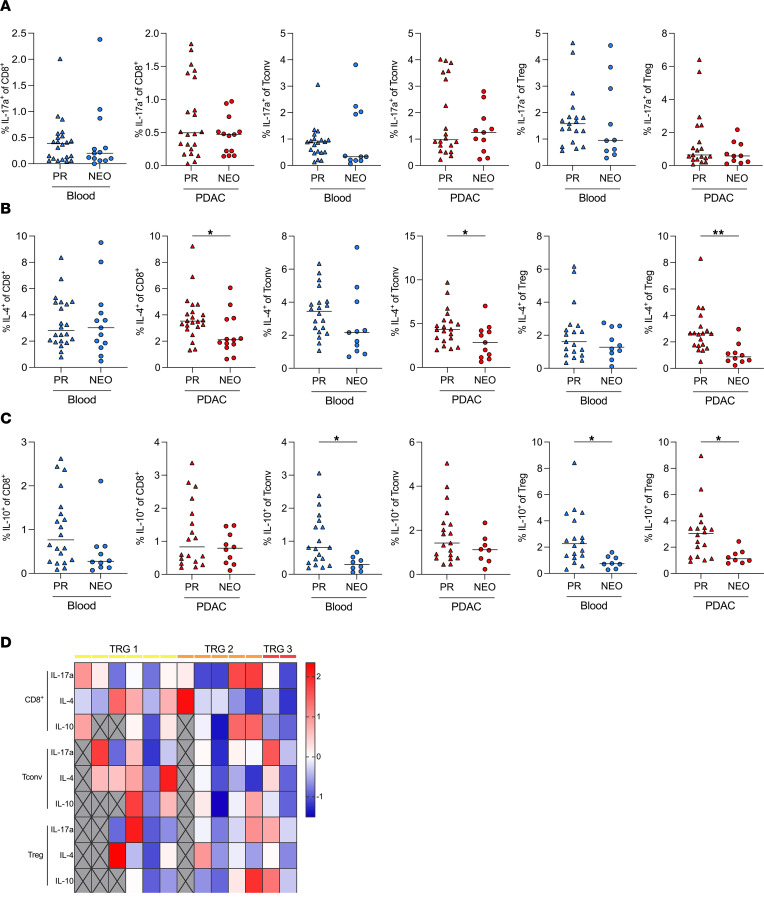
PDAC-infiltrating T cells have reduced antiinflammatory cytokine production after neoadjuvant chemotherapy. Intracellular cytokine production of circulating and matched PDAC-infiltrating T cells from patients with PDAC. Percentages of (**A**) IL-17a, (**B**) IL-4, and (**C**) IL-10 production by CD8^+^ T cells (left), CD4^+^ Tconv cells (middle), and Tregs (right). IL-17a^+^ and IL-4^+^ cells of CD8^+^ T cells, *n* = 22 PR, *n* = 13 NEO; of Tconv cells, *n* = 20 PR, *n* = 11 NEO; of Tregs, *n* = 19 PR, *n* = 10 NEO. IL-10^+^ cells of CD8^+^ T cells, *n* = 20 PR, *n* = 10 NEO; of Tconv cells, *n* = 19 PR, *n* = 9 NEO; of Tregs, *n* = 18 PR, *n* = 9 NEO. (**D**) Heatmap depicting the percentage of IL-17a–, IL-4–, and IL-10–expressing CD8^+^ T cells, CD4^+^ Tconv cells, and Tregs standardized to *z* score ordered by tumor regression grade (TRG). Missing values are shown in gray. Each point represents data from 1 patient. Medians are shown as horizontal lines. Unpaired 2-tailed *t* test. *P < 0.05.

To assess the production of cytokines that are associated with an antiinflammatory response, we stained for IL-17a, IL-4, and IL-10 (Supplemental Figure 4). All T cell subsets showed minimal IL-17a production (Figure 3A). All T cell subsets had markedly lower IL-4 production in the NEO cohort than in the PR cohort (Figure 3B). The production of IL-10 by CD8^+^ and CD4^+^ Tconv cells was generally low (Figure 3C). Tregs in the blood and tumor had reduced IL-10 production after NEO, suggestive of a lower suppressive capacity (Figure 3C). Notably, the highest expression of IL-4 was found in patients with a minor response (TRG1) to NEO (Figure 3D).

### Neoadjuvant chemotherapy decreases the proportion of functionally exhausted CD8^+^ T cells in PDAC.

**Figure 4:**
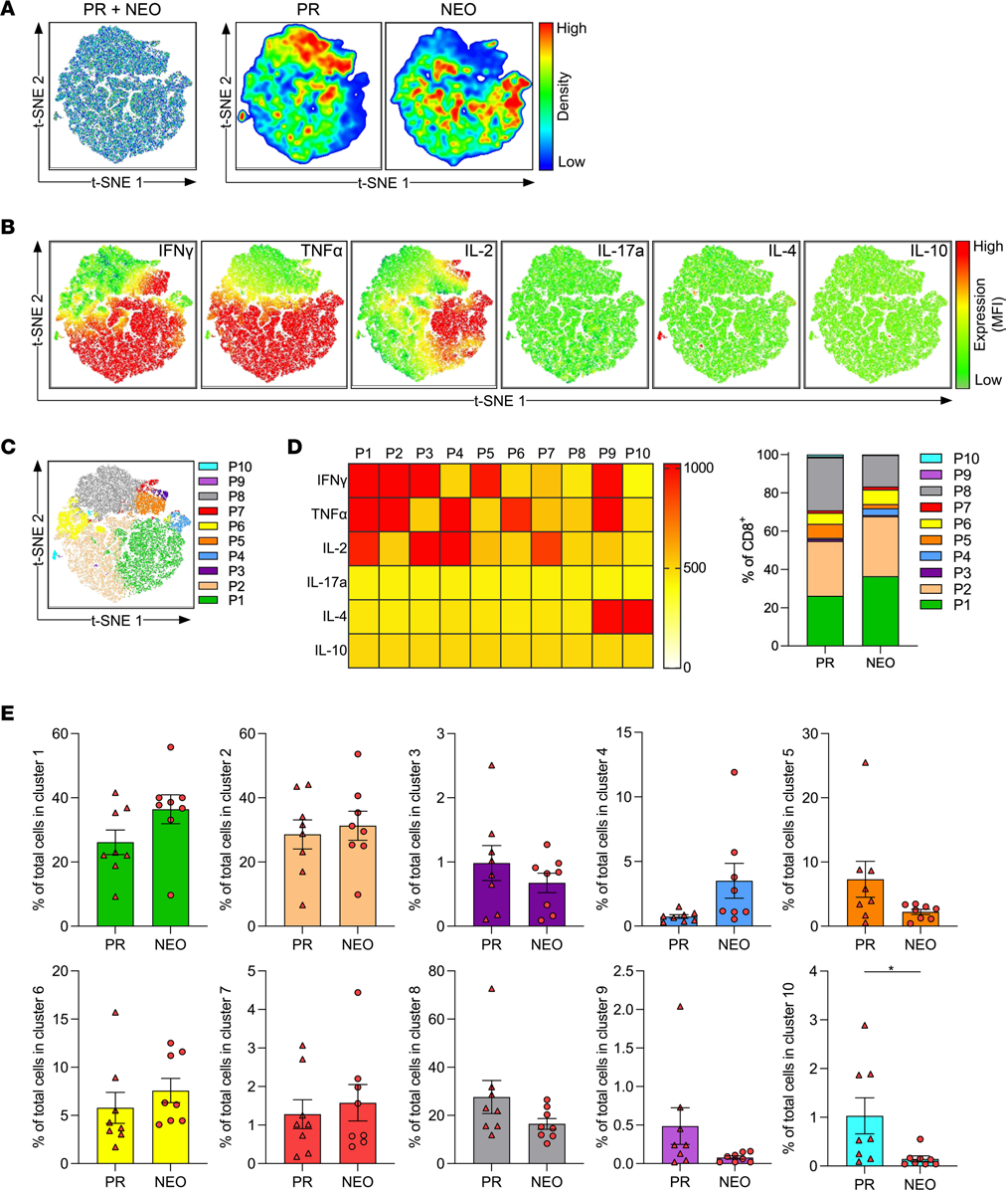
Neoadjuvant chemotherapy decreases the proportion of functionally exhausted CD8^+^ T cells in PDAC. t-SNE analysis based on intracellular cytokine expression of tumor-infiltrating CD8^+^ T cells from patients who were primary resected (PR) (*n* = 8) and patients who received NEO (*n* = 8). (**A**) t-SNE analysis of CD8^+^ T cells merged (left) and separated distribution from patients who were PR and patients who received NEO (right). (**B**) t-SNE expression of indicated cytokines. (**C**) FlowSOM clustering into 10 clusters (P1–P10). (**D**) Heatmap depicting mean fluorescence intensity for cytokine expression of each cluster (left), and bar graph showing the distribution of each cluster within PR and NEO CD8^+^ T cells (right). (**E**) Proportion of CD8^+^ T cells within indicated clusters (PR vs. NEO). Each point represents data from 1 patient. Data are shown as the mean ± SEM. Unpaired 2-tailed *t* test. *P < 0.05.

Next, we applied a t-SNE analysis on CD8^+^ T cells from patients with PR and those who received NEO (Figure 4A). Whereas the proinflammatory cytokines IFN-γ, TNF-α, and IL-2 were produced by many CD8^+^ T cells mostly simultaneously, expression of the cytokines IL-17a, IL-4, and IL-10 was rare (Figure 4B). To define populations based on the expression pattern of the different cytokines, we used FlowSOM clustering (Figure 4, C and D). A highly proinflammatory CD8^+^ T cell population (P1, as denoted in FlowSOM analysis and shown in Figure 4D), defined by the coexpression of IFN-γ, TNF-α, and IL-2, was one of the most prevalent populations (Figure 4D) and modestly higher after NEO (Figure 4E). Moreover, we found a trend toward less functionally exhausted CD8^+^ T cells that lack cytokine production in the NEO cohort (Figure 4E).

### Neoadjuvant chemotherapy increases the proportion CD4^+^ Tconv cells and Tregs with a proinflammatory profile in PDAC.

**Figure 5:**
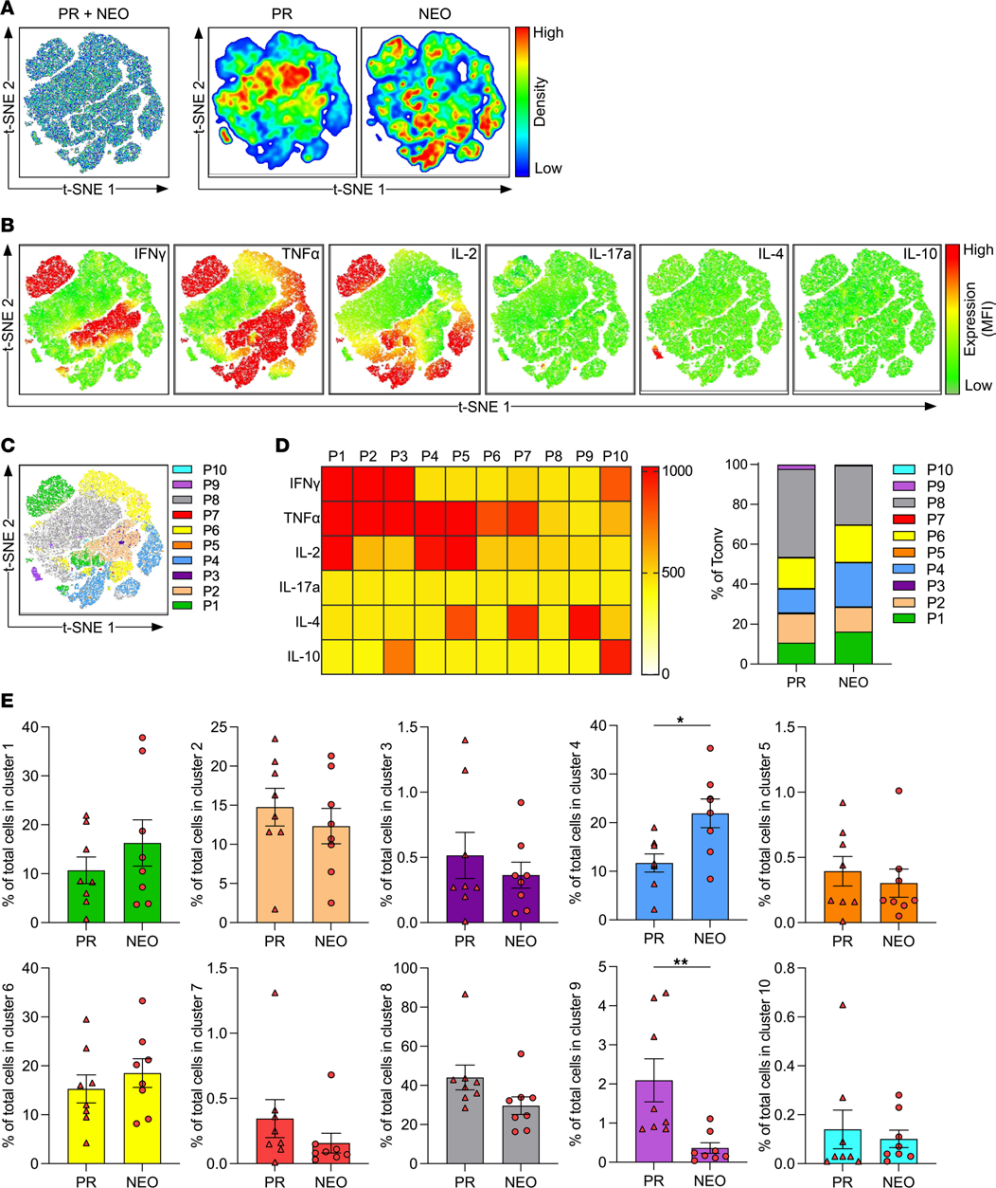
Neoadjuvant chemotherapy decreases the proportion of functionally exhausted CD4^+^ Tconv cells in PDAC. t-SNE analysis based on intracellular cytokine expression of tumor-infiltrating CD4^+^ Tconv cells from patients who were primary resected (PR) (*n* = 8) and patients who received NEO (*n* = 8). (**A**) t-SNE analysis of CD4^+^ Tconv cells merged (left) and separated distribution from patients who were PR and patients who received NEO (right). (**B**) t-SNE expression of indicated cytokines. (**C**) FlowSOM clustering into 10 clusters (P1–P10). (**D**) Heatmap depicting mean fluorescence intensity for cytokine expression of each cluster (left), and bar graph showing distribution of each cluster within PR and NEO CD4^+^ Tconv cells (right). (**E**) Proportion of CD4^+^ Tconv cells within indicated clusters (PR vs. NEO). Each point represents data from 1 patient. Data are shown as the mean ± SEM. Unpaired 2-tailed *t* test. *P < 0.05; **P < 0.01.

**Figure 6:**
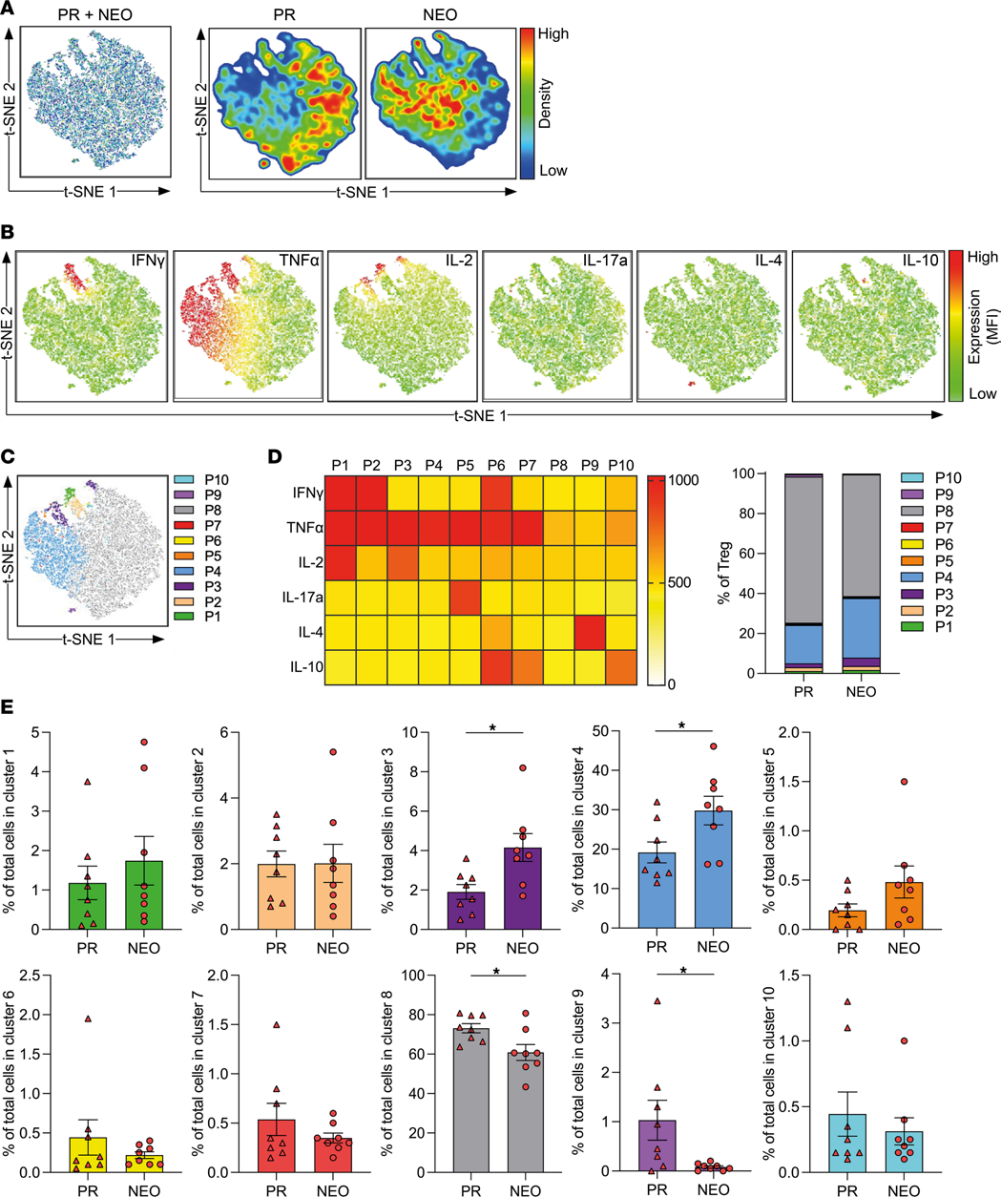
Neoadjuvant chemotherapy increases cytokine production of PDAC-infiltrating Tregs. t-SNE analysis based on intracellular cytokine expression of tumor-infiltrating Tregs from patients who were primary resected (PR) (*n* = 8) and patients who received NEO (*n* = 8). (**A**) t-SNE analysis of Tregs merged (left) and separated distribution from patients who were PR and patients who received NEO (right). (**B**) t-SNE expression of indicated cytokines. (**C**) FlowSOM clustering into 10 clusters (P1–P10). (**D**) Heatmap depicting mean fluorescence intensity for cytokine expression of each cluster (left), and bar graph showing the distribution of each cluster within PR and NEO Tregs (right). (**E**) Proportion of Tregs within indicated cluster (PR vs. NEO). Each point represents data from 1 patient. Data are shown as the mean ± SEM. Unpaired 2-tailed *t* test. *P < 0.05.

Furthermore, we performed t-SNE analysis and FlowSOM clustering on CD4^+^ Tconv cells (Figure 5, A–D) and Tregs (Figure 6, A–D). PDAC-infiltrating CD4^+^ Tconv cells consisted mostly of a population with little cytokine production (P8, as denoted in FlowSOM analysis and shown in Figure 5D). Notably, NEO markedly increased a TNF-α– and IL-2–producing population (P4, as denoted in FlowSOM analysis) and reduced mostly IL-4–producing (P9, as denoted in FlowSOM analysis) CD4^+^ Tconv cells (Figure 5, D and E). PDAC-infiltrating Tregs expressed low amounts of cytokines (Figure 6B), but 2 populations coexpressing IL-2 and TNF-α (P3, as denoted in FlowSOM analysis and shown in Figure 6D) and producing mostly TNF-α (P4, as denoted in FlowSOM analysis) were increased after NEO. The most frequent population of Tregs was characterized by little cytokine production (P8, as denoted in FlowSOM analysis), which was significantly reduced in the NEO cohort (Figure 6, D and E).

## Discussion

PDAC is a devastating disease, and improvements in survival have been marginal with current therapies over recent years. In order to develop new combinational therapies, a comprehensive understanding of the pancreatic tumor microenvironment and its modulation through current therapies is necessary. The effects of NEO on the immune landscape of PDAC have not been studied intensively to our knowledge, and, in particular, functional analysis are lacking.

In this study, we discovered immunomodulatory effects of NEO in PDAC, underlining the potential benefits of incorporating immunotherapeutic approaches in neoadjuvant treatments. We found heterogeneous and highly variable T cell infiltration across tumor specimens, consistent with a previous report (24). Notably, NEO did not affect T cell density but markedly enhanced T cell infiltration within the pancreatic ductal area. Recently, a closer proximity between antigen-experienced cytotoxic T cells and melanoma cells correlated with patient response to immune checkpoint blockade (25). Thus, NEO may promote the interaction of T cells and tumor cells. In our study, CD4^+^ T cells were the major tumor-infiltrating T cell subset, mostly consisting of CD4^+^ Tconv cells. By immunohistochemistry, it was previously shown that NEO increased the density of CD4^+^ and CD8^+^ T cells, while decreasing Treg and myeloid-derived suppressor cell frequencies (26–28). Similarly, we found a significant increase of CD4^+^ Tconv cells and a decrease of Tregs among tumor-infiltrating CD4^+^ T cells in the NEO cohort, which enhanced the ratio of CD4^+^ Tconv cells to Tregs. Increased CD4^+^ T cell frequencies in neoadjuvantly treated patients with PDAC were associated with improved survival (29). In other studies, patients receiving neoadjuvant FOLFIRINOX exhibited the densest CD8^+^ T cell infiltration (30). Especially in responders to FOLFIRINOX, a significantly decreased frequency of Tregs and increased frequency of CD8^+^ T cells was observed in the peripheral blood from patients with PDAC (31). Notably, primarily resected tumors are not the ideal control for comparison with more advanced tumors from patients who receive NEO, potentially explaining why there was no observable difference in the percentage of tumor-infiltrating CD8^+^ and CD4^+^ T cells between the PR and NEO cohort.

To our knowledge, our analyses of tumor-infiltrating T cells provide new insights into the functional profile of T cell subpopulations in PDAC. Notably, NEO decreased the proportion of exhausted T cells. CD4^+^ Tconv cells produced more IFN-γ in the tumor compared with matched peripheral blood independent of pretreatment but showed enhanced IL-2 production in the NEO cohort compared with the PR cohort. Tumor-infiltrating Tregs showed increased TNF-α and IL-2 production after NEO. Recently, we have shown that PD-1–expressing Tregs in PDAC and tumor-draining lymph nodes are associated with lymph node metastasis (32). In fact, in this study, NEO specifically reduced IL-10 production by circulating and tumor-infiltrating Tregs, suggesting a reduced suppressive capacity. Particularly, the production of the antiinflammatory cytokine IL-4 was reduced in the NEO cohort across all T cell subpopulations. Overall, NEO increased the proinflammatory function of tumor-infiltrating CD4^+^ T cells, with enhanced TNF-α and IL-2 but reduced IL-4 and IL-10 expression. This indicates a shift from an antiinflammatory Th2 phenotype toward a proinflammatory Th1 phenotype, which was previously found to be beneficial for patient survival (18). In line with our observations, chemotherapy-induced immunomodulation has also been described for tumor-associated macrophage polarization in PDAC after NEO (33). It is still unclear whether these chemotherapy-induced effects are tumor-driven or dependent on systemic host factors. However, in our study, specific changes in tumor-infiltrating but not circulating immune cells suggest dependency on tumor cell activity. Moreover, gemcitabine-induced tumor cell apoptosis may activate the immune system through the release of endogenous tumor antigens. Long-term treatment with gemcitabine has been shown to enhance antigen presentation and immune checkpoint expression in murine PDAC (34). We have previously shown that gemcitabine enhanced necroptosis in PDAC, which, in turn, promoted macrophage-induced adaptive immune suppression and tumor progression through CXCL1 and Mincle signaling (35). Moreover, in mice, both gemcitabine and 5-fluoruoracil reduced the frequency of intratumoral myeloid-derived suppressor cells (36, 37). In patients with PDAC, gemcitabine combined with recombinant cytokines and vaccines enhanced the frequency of tumor-specific T cells and resulted in objective response rates (38). Furthermore, gemcitabine has shown efficacy in combination with CD40 stimulation of T cells in eradication of established mouse tumors (39, 40). In addition, radiation led to pronounced intratumoral immune suppression via expansion of immune-suppressive tumor-associated macrophages, resulting in T cell exhaustion in PDAC (41).

In conclusion, NEO not only affects T cell frequencies in PDAC, but it also drives T cells toward a proinflammatory profile. Combining current neoadjuvant chemotherapeutic regimens with immunotherapeutic approaches is a promising strategy to improve the antitumoral effects of chemotherapy in PDAC. Including immunotherapy in the neoadjuvant setting may unleash more effective antitumor and long-term immunity in PDAC.

## Methods

### Patient samples.

The cohort for multiplex immunofluorescence consisted of 91 patients with PDAC, who underwent surgery at the Department of Visceral, Thoracic and Vascular Surgery at the University Hospital Carl Gustav Carus in Dresden, Germany, between 2008 and 2021. All tumor samples were formalin fixed and paraffin embedded, and a serial section was stained with hematoxylin and eosin for histologic evaluation by a trained pathologist. In addition, fresh tumor specimens and blood samples were obtained from patients with PDAC, who underwent surgery at the same institution between 2018 and 2022. Blood was drawn before surgical incision. A trained pathologist determined the TRG in patients after NEO according to Le Scodan (42). Clinical tumor stages were determined according to the TNM classification system (43). Clinical characteristics are shown in Supplemental Tables 1–5.

### Multiplex immunofluorescence.

Paraffin-embedded PDAC specimens were stained as described previously (43). In brief, the Opal kit, together with the Vectra 3 automated quantitative pathology imaging system (both from Akoya Biosciences) were used. Slides were stained for primary antibodies directed against PanCK (clone AE1/AE3, 1:250, Thermo Fisher Scientific), CD3 (polyclonal, 1:75, Dako) and counterstained with spectral 2-(4-amidinophenyl)-1H-indole-6-carboxamidine (DAPI, Akoya Biosciences). A trained pathologist defined the tumor area. The Phenochart and inForm softwares (both from Akoya Biosciences) were used for analysis and trained to distinguish between duct and stroma based on PanCK and DAPI expression.

### Flow cytometry.

Single-cell suspensions for flow cytometry were prepared as described previously (44). Samples were stained with monoclonal antibodies directed against CD45 (HI30), CD3 (UCHT1), CD4 (RPA-T4), CD8 (SK1; all from BD Biosciences). For FOXP3 detection, cells were fixed, permeabilized with the eBioscience Foxp3/Transcription Factor Staining Buffer Set (Thermo Fisher Scientific) after extracellular staining, and stained with anti-FOXP3 (206D, Biolegend) according to the manufacturer’s protocol. For intracellular cytokine staining, cells were stimulated with phorbol-12-myristate-13-acetate (50 ng/mL) and ionomycin (1 μg/mL) for 4 hours at 37°C, 5% CO_2_, in the presence of 1 mg/mL Brefeldin A (BD Biosciences) in RPMI medium containing 10% heat-inactivated (60°C) FCS and 1% penicillin/streptomycin (all Gibco). After stimulation, cells were washed, and extracellular staining was performed. Cells were fixed, permeabilized with the eBioscience Foxp3/Transcription Factor Staining Buffer Set and stained with monoclonal antibodies directed against IFN-γ (4S.B3), TNF-α (Mab11), IL-2 (MQ1‑17H12), IL-4 (8D4-8), IL-10 (JES3-19F1), and IL-17A (N49-653; all from BD Biosciences) according to the manufacturer’s protocol. Flow cytometry was carried out on the LSR Fortessa flow cytometer (BD Biosciences). Data were analyzed using FlowJo v10.7.1 (Treestar). The minimum number of events to gate for cytokine^+^ cells was 200 events.

### t-SNE analysis.

FlowJo v.10.7.1 was used for t-SNE analysis. CD8^+^ T cells, CD4^+^ Tconv cells, and Tregs from each sample were downsampled to 5,500, 7,500, and 2,000 cells, respectively, with FlowJo DownSample v3.3 plugin. Cells were concatenated to perform t-SNE analysis on a total of 88,000 CD8^+^ T cells, 120,000 CD4^+^ Tconv cells, and 32,000 Tregs based on the expression of IFN-γ, TNF-α, IL-2, IL-4, IL-10, and IL-17a with 3,000 iterations, 30 perplexities, 5,600 learning rate (eta), exact (vantage point tree) KNN algorithm, and Barnes-Hut gradient algorithm. In addition, FlowSOM clustering into 10 clusters was performed using the FlowJo FlowSOM v2.6 plugin, and clusters were applied to each sample to evaluate individual proportions (45). Heatmaps showing the mean fluorescence intensities of cytokines for each cluster were created with GraphPad Prism 8.0.

### Statistics.

To compare the clinicopathological characteristics of patients with PR and those who received NEO, we used the Mann-Whitney test for age distribution and Fisher’s exact test for other parameters with R software (The R Foundation, version 4.0.0). Data are shown as mean ± SEM or median. Two-tailed unpaired Student’s *t* test was applied to determine statistical significance using GraphPad Prism 8.0. *P* ≤ 0.05 was considered statistically significant. To standardize cytokine expression to *z* scores, the mean cytokine expression of the respective T cell subset was subtracted from the individual expression and divided by the SD.

### Study approval.

All surgical tumor samples and blood samples used in this study were taken from individuals treated at the University Hospital Dresden. All patients signed written informed consent, and studies were approved by the Ethics Committee of Technische Universität Dresden (EK446112017). The study was conducted in accordance with the ethical standards established by the Declaration of Helsinki.

## Author contributions

MH, LS, and AMS conceptualized and designed the study. MH, IP, JG, LM, MS, LS, and AMS developed the methodology. MH, IP, JG, LM, US, DEA, LS, and AMS acquired data. MH, IP, LM, US, DEA, MS, LS, and AMS analyzed and interpreted data. MH, IP, JG, LM, DD, CR, JVR, CK, RD, US, DEA, MS, JW, LS, and AMS wrote, reviewed, and/or revised manuscript. MS, JW, LS, and AMS provided administrative, technical, or material support. LS and AMS supervised the study.

## Supplementary Material

Supplemental data

ICMJE disclosure forms

## Version 1 11/22/2022

Electronic publication
