# Supplementary figures and images for: Neoadjuvant chemotherapy drives intratumoral T cells toward a proinflammatory profile in pancreatic cancer
Source: JCI Insight. 2022 Nov 22;7(22):e152761. doi: 10.1172/jci.insight.152761 (PMC9746809; doi:10.1172/jci.insight.152761)

A

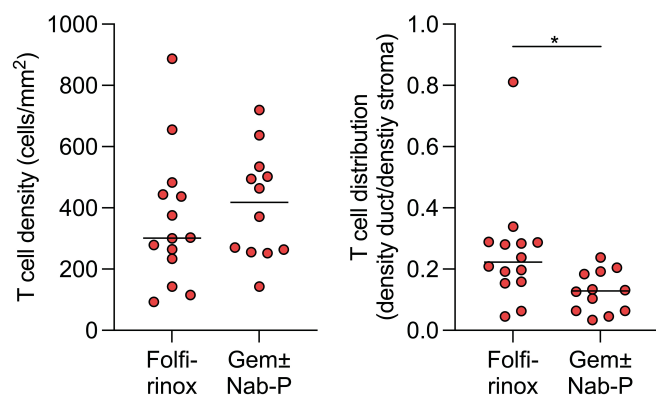

Supplement: Supplemental data [file jciinsight-7-152761-s099.pdf]
